# Differential Calcium Signaling Mediated by Voltage-Gated Calcium Channels in Rat Retinal Ganglion Cells and Their Unmyelinated Axons

**DOI:** 10.1371/journal.pone.0084507

**Published:** 2014-01-08

**Authors:** Allison Sargoy, Xiaoping Sun, Steven Barnes, Nicholas C. Brecha

**Affiliations:** 1 Department of Neurobiology and Jules Stein Eye Institute, David Geffen School of Medicine, University of California Los Angeles, Los Angeles, California, United States of America; 2 Veterans Administration Greater Los Angeles Healthcare System, Los Angeles, California, United States of America; 3 Departments of Physiology & Biophysics and Ophthalmology & Visual Sciences, Dalhousie University, Halifax, Nova Scotia, Canada; Vanderbilt University Medical Center, United States of America

## Abstract

Aberrant calcium regulation has been implicated as a causative factor in the degeneration of retinal ganglion cells (RGCs) in numerous injury models of optic neuropathy. Since calcium has dual roles in maintaining homeostasis and triggering apoptotic pathways in healthy and injured cells, respectively, investigation of voltage-gated Ca channel (VGCC) regulation as a potential strategy to reduce the loss of RGCs is warranted. The accessibility and structure of the retina provide advantages for the investigation of the mechanisms of calcium signalling in both the somata of ganglion cells as well as their unmyelinated axons. The goal of the present study was to determine the distribution of VGCC subtypes in the cell bodies and axons of ganglion cells in the normal retina and to define their contribution to calcium signals in these cellular compartments. We report L-type Ca channel α1C and α1D subunit immunoreactivity in rat RGC somata and axons. The N-type Ca channel α1B subunit was in RGC somata and axons, while the P/Q-type Ca channel α1A subunit was only in the RGC somata. We patch clamped isolated ganglion cells and biophysically identified T-type Ca channels. Calcium imaging studies of RGCs in wholemounted retinas showed that selective Ca channel antagonists reduced depolarization-evoked calcium signals mediated by L-, N-, P/Q- and T-type Ca channels in the cell bodies but only by L-type Ca channels in the axons. This differential contribution of VGCC subtypes to calcium signals in RGC somata and their axons may provide insight into the development of target-specific strategies to spare the loss of RGCs and their axons following injury.

## Introduction

Calcium is an intracellular signalling messenger that plays a central role in many physiological functions including gene expression, synaptic plasticity and cell regulation [1], [2]. Calcium signalling mediated through voltage-gated Ca channels (VGCCs), other calcium permeable channels, and intracellular stores, plays a key role in mediating cell degeneration following injury [3]. Unregulated elevated calcium signalling has been implicated in the degeneration of retinal ganglion cells (RGCs) in numerous injury models including those for ischemia, optic nerve trauma and elevated IOP [4]. Owing to its dual roles in maintaining homeostasis and triggering apoptotic pathways in healthy and injured cells, respectively, investigation of VGCC regulation of intracellular calcium as a potential strategy to reduce the loss of RGCs is warranted. Due to the anatomical accessibility of RGCs and their axons, the retina provides an advantageous system in which to investigate the mechanisms of calcium signalling in neurons and their unmyelinated axons within the eye. The goal of the present study was to describe the distribution of VGCC subtypes and their contribution to the calcium signal in ganglion cells bodies and axons in the normal retina, which provides a foundation for understanding RGC Ca^2+^ signalling and the cellular response to injury.

VGCCs are transmembrane, multimeric proteins comprised of a pore forming α1 subunit that is typically associated with auxiliary α2δ and β subunits. The α1 subunit functions as the voltage sensor and establishes the biophysical and pharmacological properties of the channel [5]. The predominantly extracellular α2δ and intracellular β subunits enhance trafficking and expression of the Ca channel α1 subunits to the plasma membrane [5], [6] and also alter the biophysical properties of the channel [7]–[9]. Ten mammalian genes have been identified that encode the α1 subunit, while four genes have been identified that encode the α2δ and β subunits each [5]. VGCCs are also classified by electrophysiological and pharmacological properties, which give rise to L-, N-, P/Q-, R- and T-type Ca channel subtypes. Although physiological evidence has suggested that mammalian RGC somata express all of these Ca channel subtypes [10]–[16], questions still remain regarding the distribution of VGCCs and their contribution to calcium signalling in both ganglion cell bodies and their axons.

We report differential expression of α1 subunits for L-, N-, and P/Q-type Ca channels in rat RGC somata and their axons. While immunostaining for T-type Ca channel α1 subunits was not possible due to a lack of selective reagents in rat RGSs, we patch clamped isolated RGCs and showed the presence of T-type Ca channels in RGC somata. Calcium imaging of RGCs showed that subtype specific Ca channel antagonists reduced depolarization-evoked calcium signals mediated by L-, N-, P/Q- and T-type Ca channels to different degrees in the cell bodies and axons. The differential expression and contribution of VGCC subtypes to calcium signals in RGC somata and their axons may provide insight into the development of target-specific strategies to spare the loss of RGCs and their axons following injury.

## Methods

Immunohistochemical, patch clamp and calcium imaging experiments were performed at UCLA in accordance with the guidelines for the welfare of experimental animals issued by the U.S. Public Health Service Policy on Human Care and Use of Laboratory Animals (2002). The University of California-Los Angeles Animal Research Committee approved this study and the institution where the studies were undertaken is authorized to house and sacrifice animals for research purposes. Male and female adult Sprague-Dawley rats (Charles River Lab, Wilmington, MA) between the age of 3–5 weeks were deeply anaesthetized with 1–3% isofluorane (IsoFlo, Abbott Laboratories), decapitated and the eyes removed.

### Immunohistochemical labelling

Following removal of the anterior chamber and lens, the eyecups were fixed in 4% (w/v) paraformaldehyde in 0.1 M phosphate buffer (PB), pH 7.4, for 15–60 min at room temperature, washed in 0.1 M PB and cryoprotected in 30% sucrose overnight at 4°C. Vertical sections (12 µm) were cut using a Leica CM3050S cryostat (Leica Microsystems, Buffalo Grove, IL) and mounted onto gelatin-coated slides.

Frozen retinal sections were thawed for 3 min at 37°C on a tissue-warming tray and rinsed in 0.1 M PB (pH 7.4) three times for 10 min each. Retinal sections were incubated in a blocking solution containing 10% normal goat serum, 1% bovine serum albumin, 0.5% Triton X-100 in 0.1 M PB for 1 h at room temperature. The primary antibodies were diluted in 3% normal goat serum, 1% bovine serum albumin, 0.5% Triton X-100 in 0.1 M PB, pH 7.4, for 12–16 h at room temperature. Secondary antibodies conjugated with Alexa Fluor −488, −568 or −633, anti-rabbit, -guinea pig or -mouse (1∶1000; Invitrogen, Carlsbad, CA) were applied for 1 h at room temperature. Images were acquired on a Zeiss Laser Scanning Microscope 510 Meta or 710 (Carl Zeiss, Inc., Thornwood, NY) using a Zeiss C-Apochromat 40× (1.2 NA) corrected water objective. Images were further processed in Adobe Photoshop CS5 v12.1 (Adobe Systems, San Jose, CA) to improve contrast levels.

**Figure 1:**
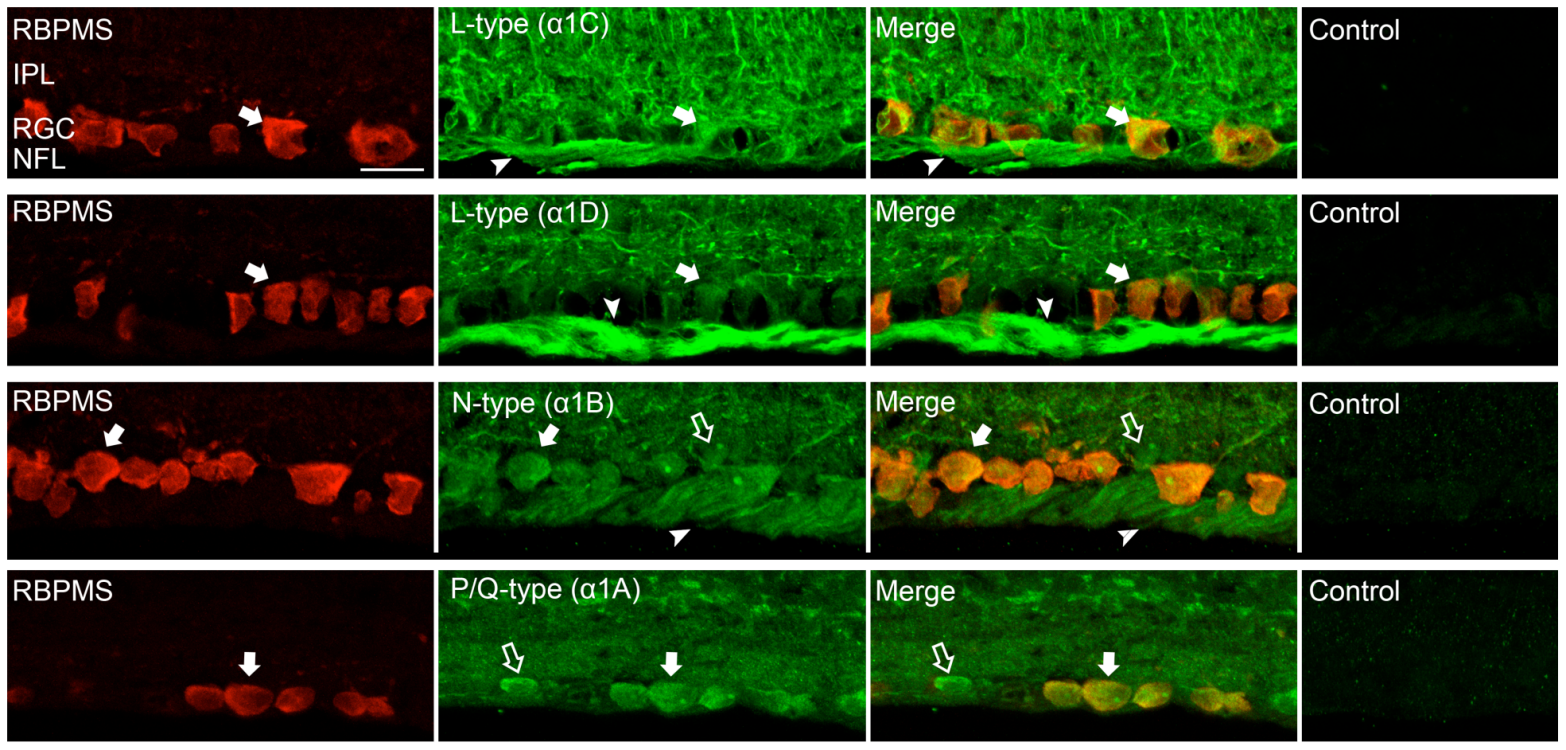
Expression of L-, P/Q-, N-type Ca channels in the proximal retinal. Ca channel α_1_ subunits co-express with the ganglion cell antibody RBPMS in vertical sections showing the inner plexiform layer (IPL), the ganglion cell layer (GCL) and the nerve fiber layer (NFL). (**Top row**) Double immunostaining with α1C subunit and RBPMS antibodies shows colocalization in the RGC somata (filled arrow). RGC axons (arrowhead) as well as putative Müller and neuronal cell processes in the IPL showed immunostaining. Control preadsorption of α1C antibody with the immunization peptide showed no staining. (**Second row**) Double immunostaining with α1D subunit and RBPMS antibodies shows colocalization in the RGC somata (filled arrow). Numerous processes in the IPL as well as the RGC axons throughout the NFL (arrowhead) were also labelled. Control preadsorption of α1D antibody with the immunization peptide showed no staining. (**Third row**) Double immunostaining with α1B subunit and RBPMS antibodies shows colocalization in RGC somata (filled arrow). Processes in the IPL, putative displaced amacrine cells in the GCL (open arrows) and RGC axons throughout the NFL (arrowheads) showed immunostaining. Control preadsorption of α1B antibody with the immunization peptide showed no staining. (**Bottom row**) Double immunostaining with α1A subunit and RBPMS antibodies shows colocalization in the RGC somata (filled arrow). Labelling in the IPL and in putative displaced amacrine cells in the GCL (open arrows) was also seen. Control preadsorption of α1A antibody with the immunization peptide showed no staining. Scale bar is 20 µm.

Omission of the primary antibody was performed as a negative control to test for non-specific binding by the secondary antibody. Preadsorption controls were performed to evaluate the specificity of primary antibody immunostaining. In brief, the α1D and α1A antibodies were diluted with their corresponding antigen (RS Systems; immunogen sequence DNKVTIDDYQEEAEDKD and RDPDARRAWPGSPERAPGREGPYGRESEPQQRE, respectively) at a final concentration of 1 µg/ml for 12–16 h overnight at 4°C. Preadsorption controls were also performed with the α1C and α1B antibodies (Alomone; ACC-003 and ACC-002, respectively) at a final concentration of 1 µg/ml for 12–16 h overnight at 4°C. Immunostaining was absent in vertical retinal sections incubated with the preadsorbed antibody (Fig. 1).

#### Antibodies

The following polyclonal antibodies were used to obtain staining of the α1 subunits: α1C (Cav1.2, Alomone, ACC-003, 1∶1000, Jerusalem, Israel), α1D (Cav1.3, Sigma, C1728, St. Louis, MO, 1∶100), α1A (Cav2.1, Synaptic Systems, 152–103, 1∶7500, Göttingen, Germany), α1B (Cav2.2, Alomone, ACC-002, 1∶1000, Jerusalem, Israel). A neurofilament-medium (NF-M) (Millipore, MAB1621, Billerica, MA) antibody was used for the detection of RGC axons. A guinea pig polyclonal antibody was generated against the N-terminus of the RNA Binding Protein Multiple Splice (RBPMS) polypeptide (RBPMS4-24), GGKAEKENTPSEANLQEEEVR, by a commercial vendor (ProSci, Poway, CA). RBPMS is highly conserved among mammals and the polypeptide sequence used for immunization is identical in mouse, rat, monkey and human (NCBI Protein Bank, http://www.ncbi.nlm.nih.gov/protein). Guinea pig sera were collected following immunization and affinity purified using a RBPMS polypeptide affinity column. The affinity purified antibody was shown to immunostain ganglion cells in mouse and rat retina (Rodriguez et al., 2013, submitted). To evaluate the specificity of the RBPMS immunostaining, a preadsorption control was performed with the rabbit antibody. Briefly, the RBPMS antibody was diluted in 0.1 M PB containing 0.5% Triton X-100 and mixed with the RBPMS polypeptide at a final concentration of 1 µg/ml for 2 h at room temperature. No RBPMS immunostaining was present in tissue sections incubated with the preadsorbed rabbit antibodies to RBPMS and processed by standard immunohistochemical techniques.

Specificity of the rabbit polyclonal antibody against the α1A subunit was previously confirmed by Western blotting in α1A knockout mice [17]. The rabbit antibody against the α1B protein was shown to detect a 200 kDa band in a Western blot of rat brain homogenates [18]. The specific band for the α1B protein was absent in Western blots incubated with the control antigen. The rabbit polyclonal antibody against the α1C protein was shown to detect major and minor bands in a Western blot of human cardiac tissue homogenates [19]. The rabbit polyclonal antibody against the α1D subunit has been shown to recognize a band corresponding to Cav1.3 α1 subunit in Western blot of rat brain homogenates [20]. Preadsorption with the control antigen eliminated the band.

### Patch clamp recording from isolated ganglion cells

Retinas were isolated in Ca^2+^ and Mg^2+^ free Hank's Balanced Salt Solution (HBSS; HyClone) and transferred into 10 ml Hibernate A medium containing 126 units/ml deoxyribonuclease and 1.5 mg/ml Bovine serum albumin (BSA) following incubation in 13 units/ml papain (Worthington) dissolved in Ca^2+^- and Mg^2+^-free HBSS at 37°C for 30 minutes. The enzymatically treated retinas were gently triturated with a fire-polished Pasture pipette and then plated on to glass coverslips, which were pre-coated with 0.1 mg/ml Con A. Cells adhered and were ready for patch clamping 1 h after plating.

Ca channel currents were measured under whole-cell voltage clamp using patch electrodes fabricated from borosilicate glass (0.86/1.5 mm in inner/outer diameter) with a Flaming-Brown horizontal puller (P-87, Sutter Instrument Co, Novato, CA). After filling, electrodes had a final resistance of 5–7 MΩ. Patch electrodes were filled with internal solution containing (mM): 130 K gluconate, 20 KCl, 2 MgATP, 0.5 Na_2_GTP, 10 Na phosphocreatine, and 1 EGTA, at pH 7.2. Cells with access resistance of <25 MΩ were used. Capacitance and series resistance were optimized and compensated by ∼60%. Current recordings were not corrected for linear leakage resistance. Cell membrane capacitance (*C*_m_) was between 6–12 pF. The bath solution contained (in mM): 10 CaCl_2_, 140 TEACl, 1 MgCl_2_, 10 HEPES, 1 µM TTX, at pH 7.3, or 10 BaCl_2_, 130 NaCl, 10 TEACl, 5 CsCl, 1 MgCl_2_, 10 HEPES, and 1 µM TTX, at pH 7.3. Voltage clamp experiments were performed at room temperature with an Axopatch 200B (Axon Instruments, Union City, CA). Pulse generation, data acquisition and analysis were done with a PC equipped with a Digidata 1322A analog-to-digital (A/D) interface in conjunction with Clampex 9.0 programs (Axon Instruments). Currents were filtered with a 4-pole Bessel Filter at 5 kHz. The holding potential was −70 mV.

### Calcium imaging in wholemount retinas

Following enucleation, Fluo-4 pentapotassium salt (0.5 µl of 40 mM stock in H_2_O) was injected into the optic nerve stump approximately 1 mm posterior to the globe. To enable retrograde labelling of RGCs and RGC axons, the eyeball was placed in mammalian saline solution bubbled with 95% O_2_/5% CO_2_ for 1 h at room temperature in the dark. The retina was then isolated from the eyecup and divided into quadrants. One quadrant was mounted on a glass slide using a harp slice grid (ALA Scientific, HSG 5DD, Farmingdale, NY).

An eight channel gravity superfusion system (ALA Scientific, Farmingdale, NY) delivered solutions containing (in mM): 120 NaCl, 3 KCl, 2 CaCl_2_, 1.20 NaH_2_PO_4_, 1 MgCl_2_, 25 NaHCO_3_, and 10 glucose bubbled with 95% O_2_/5% CO_2,_ to the retinal preparation. RGCs and RGC axons were depolarized by raising the [K^+^]_o_ from 3 mM to 60 mM for 33 s to activate VGCCs. Na^+^ was reduced by 57 mM to maintain isosmolarity in the elevated K^+^ solution. K^+^ pulses were always applied in pairs with ∼680 s separation. Images were acquired at 5 s intervals with a Zeiss LSM 5 Pascal laser scanning microscope equipped with a water-immersion Axoplan 40× (NA 0.8) objective. Excitation was provided by the 488 nm line of the argon laser while collecting emission through a 505 nm long-pass filter. Fluorescence intensity values were acquired by placing regions of interest (ROIs) on specific RGC somata and axon bundles.

#### Analysis

Fluorescence intensity changes were always recorded in response to paired high K^+^ pulses with the change produced by the second high K^+^ induced peak normalized to the change produced by the first high K^+^ peak. In all cases, control values were first recorded with paired K^+^ pulses with no drug and in cell bodies, these typically showed a modest reduction (a few percent) of the second peak relative to the first. Then, for testing of VGCC or Na channel blockers, drugs were applied only during the second K^+^ pulse of a pair, whose peak value was normalized against the first peak value. Thus, for each drug change value considered, the data were taken as the amplitude of the second K^+^ peak divided by that of the first, and these values were analysed with respect to their matching controls derived from paired K^+^ peaks measured in the absence of drug. Experiments were performed on wholemount retinas from 3–5 rats for each drug. ROIs corresponding to individual RGC somata or axon bundles were averaged in every trial, each considered an independent observation for statistical testing using Student's unpaired *t*-test with *p*<0.05 considered significant. The value ‘n’ equals the number of independent trials, with which value averaged from 20–90 individual RGC somata or 2–12 RGC axon bundles. Variability is reported as standard error of the mean. Figures were processed using GraphPad Prism 4.0 (GraphPad Software, Inc, La Jolla, CA). In the figures, fluorescence traces are shown normalized to the first of the paired responses to elevated K^+^ application.

#### Drugs and Chemicals

All chemicals and reagents, unless otherwise noted, were obtained from Sigma-Aldrich (St. Louis, MO). Verapamil, nifedipine, and mibefradil came from Tocris (Bristol, UK), ω-agatoxin-IVA was from Abcam (Cambridge, MA) and fluo-4 pentapotassium salt came from Invitrogen (Carlsbad, CA).

## Results

### Differential L-, N-, and P/Q-type Ca channel localization in RGC somata and their axons

To investigate the localization of VGCCs in RGCs, we immunostained rat retinal sections with RBPMS, a robust marker of RGCs [21], [22] in conjunction with specific antibodies against the α1C, α1D, α1B and α1A VGCC subunits. Double immunolabelling with RBPMS antibodies and the corresponding VGCC subunit antibodies confirmed localization of the α1C, α1D, α1B and α1A subunits to RGCs (Fig. 1). In addition, antibodies against the L-type VGCC subunits, α1C (Fig. 1, top row) and α1D (Fig. 1, second row) revealed immunolabelling in the nerve fiber layer (NFL), which is composed of fascicles of RGC axons and glial cells. The α1C subunit antibody also immunostained radial processes likely to be Müller cell processes, as previously described in chicken retina [23].

**Figure 2:**
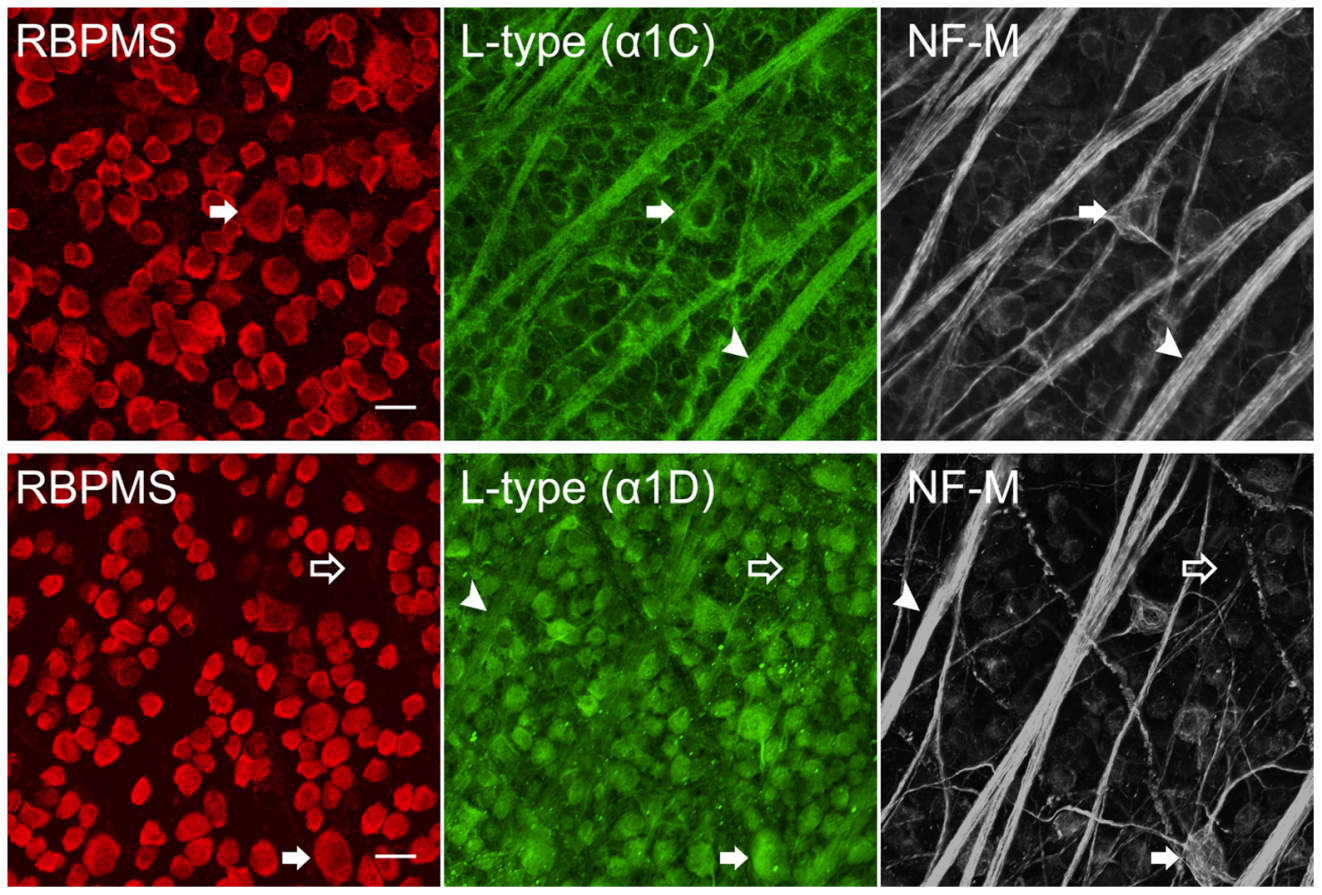
Localization of L-type Ca channels in rat retina. (**Top row**) Rat wholemount retina triple labelled with RBPMS (red), α1C VGCC subunit (green), and NF-M (white) antibodies. α1C colocalized with RBPMS, which labels RGC somata (filled arrow), and NF-M, which labels RGC axons (arrowhead), as well as putative Müller cell endfeet. Stack of five z-axis optical sections each of 0.3 µm thickness. (**Lower row**) Rat wholemount retina labelled with RBPMS (red), α1D VGCC subunit (green), and NF-M (white) antibodies. α1D was localized to RGC somata (RBPMS) (filled arrow), RGC axons (NF-M) (arrowhead) and putative displaced amacrine cells (open arrow). Stack of four optical sections each of 0.3 µm thickness. Scale bar is 20 µm.

To determine if all RGCs in the ganglion cell layer (GCL) express the α1C and α1D subunits, we performed double immunostaining in wholemount retinas, which showed colocalization of L-type VGCC subunits with all RBPMS-positive RGCs (Fig. 2). Immunoreactivity of the α1C and α1D subunits was also localized to cell bodies in the GCL that did not show immunoreactivity for the RBPMS antibody. Due to the size of the cell somata (∼10 µm), these neurons are likely to be displaced amacrine cells [24], [25].

To examine the colocalization of the α1C and α1D subunits to RGC axons of the nerve fiber layer (NFL), we performed triple immunostaining in wholemount retina with antibodies against the α1C or α1D proteins together with antibodies against RBPMS proteins and neurofilament-medium (NF-M), a marker of the neuronal cytoskeleton of RGCs and RGC axons [26], [27]. NF-M is also a marker for the large melanopsin-positive M2 cells [28]. Triple labelling revealed extensive colocalization of neurofilament with the α1C and α1D Ca channel subunits.

**Figure 3:**
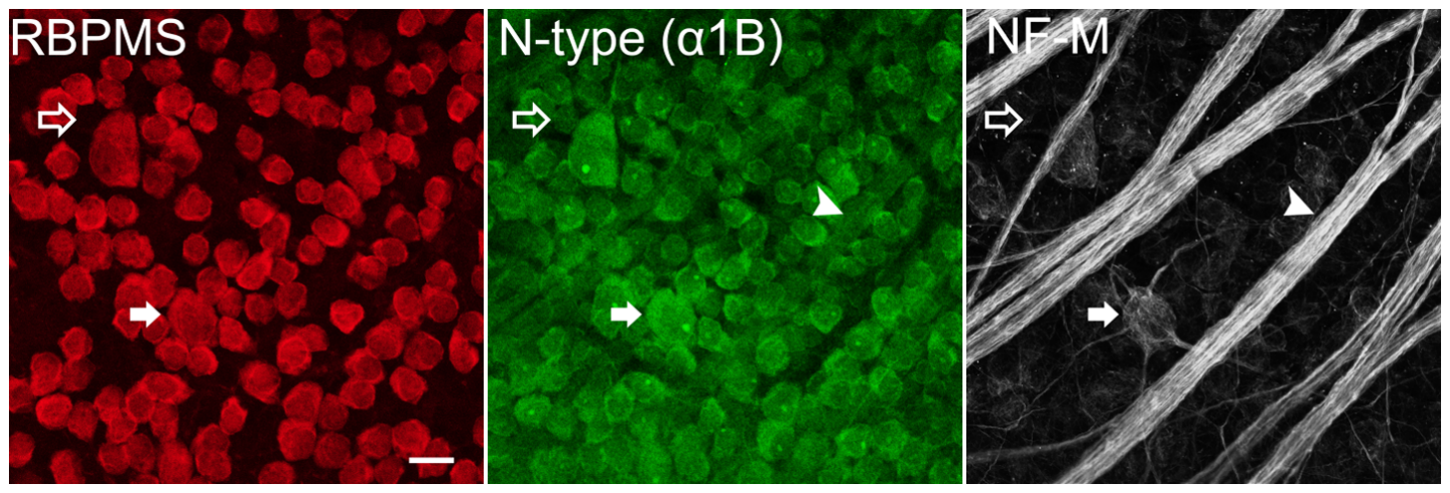
Localization of N-type Ca channels in ganglion cells in rat retina. **R** at wholemount retina labelled with RBPMS (red), α1B VGCC subunit (green), and NF-M (white) antibodies. α1B colocalized with RBPMS in RGC somata (filled arrow), NF-M in RGC axons (arrowhead) and putative displaced amacrine cells (open arrow). Stack of six optical sections each of 0.3 µm thickness. Scale bar is 20 µm.

We also performed immunohistochemical analysis of N-type Ca channels in RGC somata and their axons in vertical sections and wholemount retina. Antibodies against the N-type VGCC α1B subunit revealed immunolabelling in most RGC somata, the IPL and in the NFL (Fig. 1, third row). To determine if all RGCs in the GCL express the α1B subunit, we performed double immunostaining for the α1B Ca channel subunits and RBPMS-positive RGCs (Fig. 3). As seen with the L-type Ca channel subunits, some immunoreactivity of the α1B subunit was localized to cell bodies in the GCL that did not show immunoreactivity for the RBPMS antibody. These cell bodies are ∼10 µm in diameter, consistent with their identity as displaced amacrine cells. Triple labelling revealed colocalization of NF-M with the α1B Ca channel subunits, indicating localization of N-type Ca channels to the axons.

**Figure 4:**
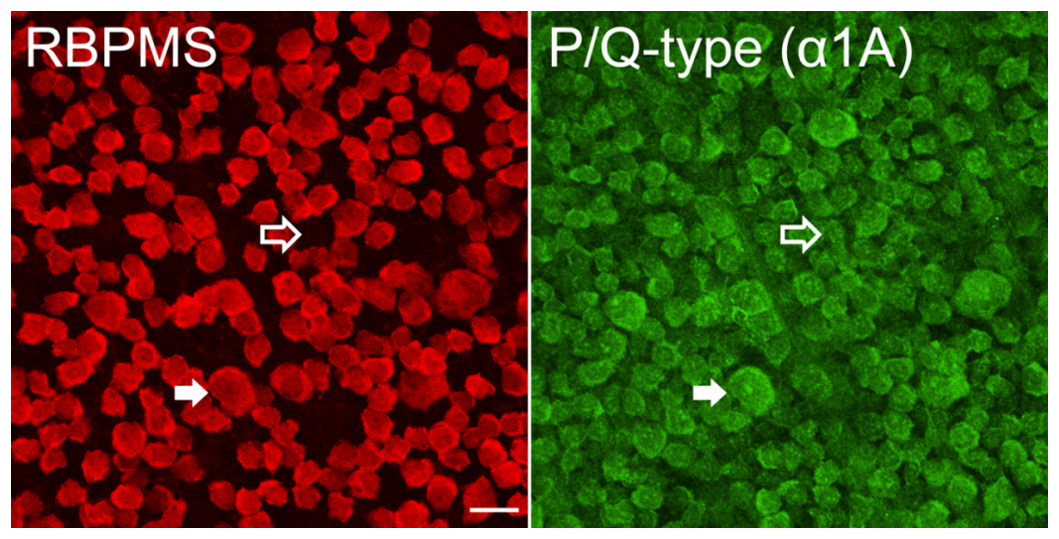
P/Q-type Ca channel expression in ganglion cells in rat retina. Retina labelled with RBPMS (red) and α1A VGCC subunit (green) antibodies. α1A was colocalized with RBPMS in RGC somata (filled arrow). Additional cell bodies in the GCL staining for α1A and not RBPMS are likely to be displaced amacrine cells (open arrow). Scale bar is 20 µm. Stack of 10 optical sections each of 0.3 µm thickness.

Antibodies against the P/Q-type VGCC α1A subunit revealed immunolabelling in RGC somata but not in the NFL (Fig. 1, bottom row). Immunohistochemical analysis of P/Q-type Ca channels in RGC somata and their axons in wholemount retina is shown in Figure 4. Double immunostaining revealed colocalization of P/Q-type VGCC α1A subunits with RBPMS-positive RGCs. Triple staining with antibodies against NF-M was not successful.

### Patch clamp recording shows the presence of T-type Ca channels in RGC somata

**Figure 5:**
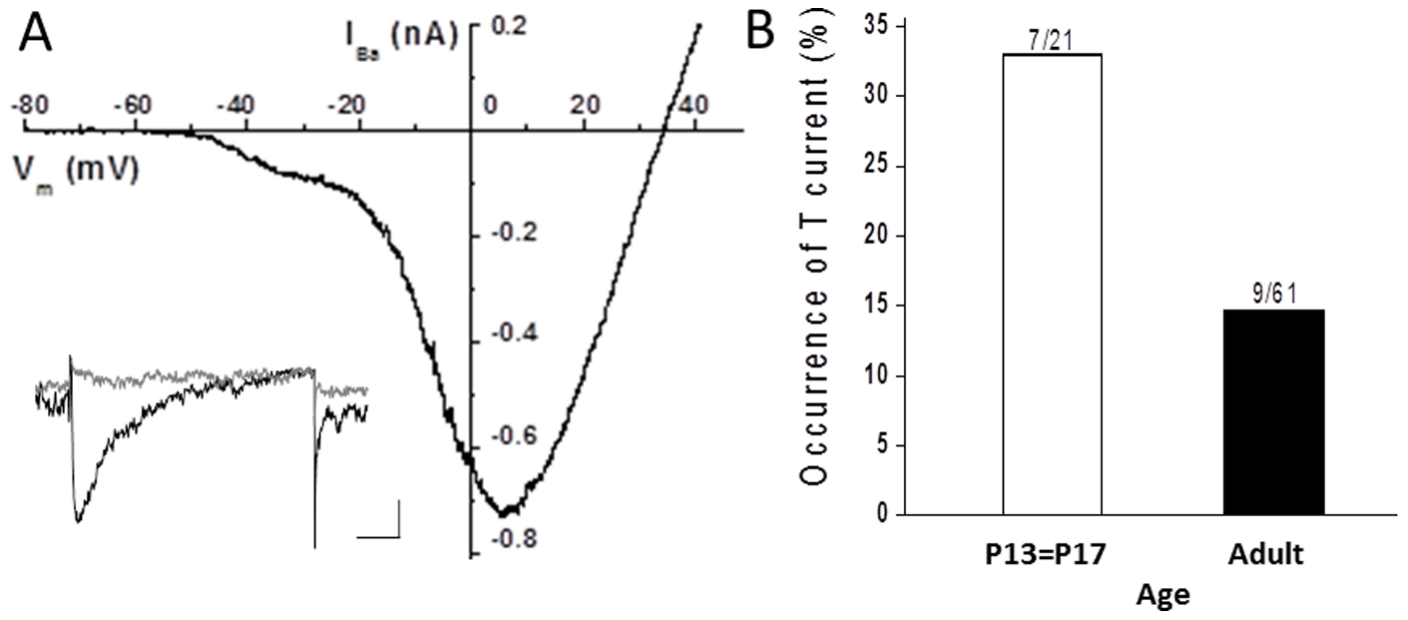
Patch clamp analysis of T-type Ca channels in isolated ganglion cells from rat retina. **A**. Current-voltage relation obtained from voltage-clamped whole cell currents in 10 mM Ba^2+^ in response to a voltage ramp from a holding potential of −120 mV. Inset shows currents recorded in 10 mM Ca^2+^ at a test potential of −30 mV from holding potentials of −90 mV (black trace) or −50 mV (grey trace). The bath solution contained 1 µM TTX. Scale bar (inset) shows 20 pA and 50 ms. **B**. The rate of occurrence of T-type Ca channel currents declines with age. Over 30% of RGCs recorded from P13–P17 rats expressed T-type Ca channel current and this rate was reduced by more than 50% in rats 4 weeks or older (adult).

To confirm the presence of T-type Ca channels in rat RGCs, we patch clamped isolated cells and used voltage clamp stimulus paradigms designed to detect the presence of T-type Ca channels. Stimulated with voltage ramps in solutions containing Ba^2+^ and TTX to enhance Ca channel currents and block inward Na channel currents, a shoulder on the negative side of the typical N-shaped whole cell current voltage relation was often recorded, as exemplified in Figure 5A. Such a shoulder is considered characteristic of T-channel activity [29]. In addition, by removing voltage-dependent inactivation with large negative holding potentials, step voltage clamp stimuli elicited transient, inactivating inward currents, as shown in the Figure 5 inset, as would be expected from the activation of T-type Ca channels. T-type Ca channels were found in RGCs at a maximum of ∼30% in young (P17–P21) rats and at a minimum of about 15% in adult rats (>4 weeks of age).

### Similar time course of calcium signals in RGCs and their axons

**Figure 6:**
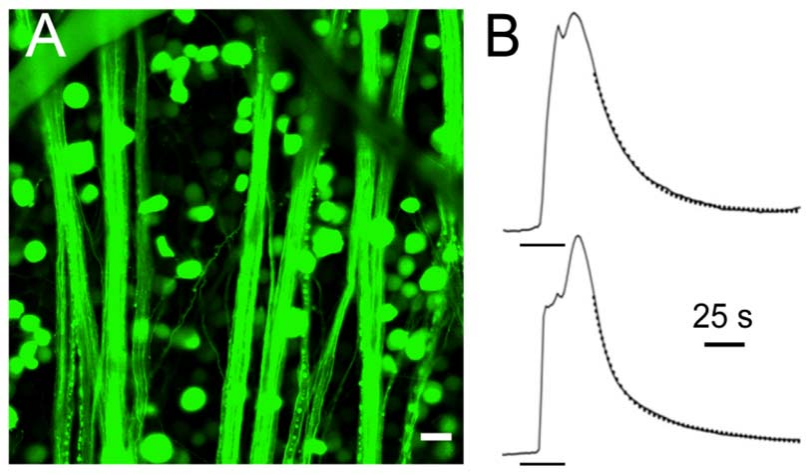
Depolarization-induced calcium signals in RGCs and their axons. **A**. Multiple RGC somata and axon bundles in fluo-4 labelled retinal wholemount. Scale bar is 20 µm. **B**. A 33 s application of 60 mM K^+^ was applied at the time indicated (bar below the rising phases of the traces) and the simultaneous responses of 23 RGC somata (lower panel) and 13 RGC axon bundles (upper panel) was recorded and fit with exponential functions for comparison. Both the axon bundle and the cell body responses were complex with sequential and unsynchronized rising phases but both the axon bundle and somatic responses relaxed with similar time courses. The falling phase of the axon bundles was best fit with a single exponential having a time course of 25.7 s (overlay trace in dots) while that of the cell body required a double exponential fit with time constants of 11.5 and 65.8 s (overlay trace in dots).

Figure 6A shows fluo-4 labelling of RGC somata and axon bundles in wholemount retina. Transient increases in [Ca^2+^] levels during depolarization with elevated K^+^ pulses, averaged and normalized from recordings made simultaneously in the RGC somata and axons, are shown in Figure 6B. Data averaged from 24 RGC somata (lower panel) and 16 axon bundles (upper panel) indicate that the increase in mean fluorescence intensity in response to the depolarizing stimulus followed a similar time course in the axons and the somata. A 33 s pulse of 60 mM K^+^ produced a rapid increase in [Ca^2+^] levels, followed by a slower falling phase. The rising phase of the response at the cell body was complex in this example, showing several transient rising phases leading to a peak at 28.3 s. The falling phase of the calcium response in the cell bodies was best fit by a double exponential time course with time constants of 11.5 s and 65.8 s. In the axons, the rising phase of the response at the cell body was likewise complex with a peak in 25.5 s, and the falling phase was well fit with a single exponential having a time constant of 25.7 s. If the calcium signals in the unmyelinated axons resulted from diffusion of cytosolic calcium from their cell bodies, slower rising and falling phases of calcium concentration would have been observed due to the delay caused by the diffusion process. Previous measurements of photolytically stimulated Ca^2+^ diffusion obtained in RGC axons of the myelinated mouse optic nerve show that the length constant for Ca^2+^ diffusion is on the order of 30 µm [30]. The regions of axons from which we recorded calcium signals varied in distance from their cell bodies, with some in excess of 500 µm, making it unlikely that the rapid, simultaneous increase of [Ca^2+^]_i_ in the axons was due to diffusion of the calcium signal generated in the somata and suggest that local sources of calcium influx are present throughout the axon, as supported by the immunohistochemical data presented above.

### Block of calcium signals induced by depolarization of RGC somata in wholemount retina

Electrophysiological studies have provided pharmacological and biophysical evidence for L-, N-, P/Q- and T-type VGCCs expression in rat RGCs [11], [14]. Our calcium signalling studies extend these earlier results providing pharmacological evidence for the presence of L-, N-, P/Q- and T-type Ca channels, differentially expressed in RGCs and their axons.

**Figure 7:**
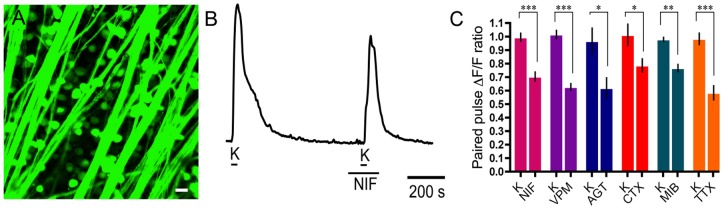
Many VGCC subtypes contribute to calcium signalling in ganglion cell bodies. A. Fluo-4 labelling of RGC somata in the wholemount retina. Scale bar is 20 µm. B. Application of nifedipine (NIF; 10 µM), an L-type Ca channel antagonist, reduced the second high K^+^-evoked calcium signal. C. Summary of Ca^2+^ imaging results in RGC somata showing the following changes in paired pulse Ca^2+^ signal in response to drugs (applied during the second K^+^ pulse) compared to their control paired K^+^ pulses (K): 10 µM nifedipine (29%±7%; p = 0.0003; n = 20), 100 µM verapamil (VPM; 39%±5%; p<0.0001; n = 17), 400 nM ω-agatoxin IVA (AGT; 35%±14%; p = 0.0364; n = 9), 3 µM ω-conotoxin-GVIA (CTX; 23%±10%; p = 0.0423; n = 15), 3 µM mibefradil (MIB; 21%±6%; p = 0.0011; n = 16) and 200 nM TTX (40%±9%; p = 0.0004; n = 14).

Figure 7A shows fluo-4 labelling of RGC somata in wholemount retina, which enabled evaluation of increased intracellular [Ca^2+^] levels during depolarization with 60 mM K^+^ pulses. Superfusion of RGCs with 60 mM K^+^ has been shown to depolarize the cells to ∼−20 mV and to increase intracellular [Ca^2+^] from resting levels (∼100 nM) up to ∼1 µM [31]. Back-labelling from the nerve stump injection site prevented labelling of displaced amacrine cells, which make up ∼60% of the cells in the ganglion cell layer in the mammalian retina [32]. Calcium imaging of RGCs in wholemount retina demonstrated that superfusion of high K^+^ (60 mM) elicited changes in the mean fluorescence. Control paired K^+^ pulses showed a reduction of the second pulse amplitude that was on average 99% of the first. Application of selective Ca channel blockers reduced the second K^+^-evoked [Ca^2+^]_i_ relative to control, as exemplified in Figure 7B where the effects of nifedipine are shown.

To determine the contribution of L-type VGCCs to the depolarization-evoked [Ca^2+^]_i_, we applied the L-type VGCC blockers nifedipine (10 µM) and verapamil (100 µM). Nifedipine and verapamil reduced the second of paired high K^+^ pulses in the RGCs by 29%±7% (p = 0.0003; n = 20) and 39%±5% (p<0.0001; n = 17), respectively (Fig. 7C). The application of the P/Q- and N-type VGCC blockers, ω-agatoxin IVA (400 nM) and ω-conotoxin-GVIA (3 µM), reduced the K^+^-evoked calcium signal by 35%±14% (p = 0.0364; n = 9) and 23%±10% (p = 0.0423; n = 15), respectively (Fig. 7C). To estimate the contribution of T-type VGCCs to the high K^+^-evoked calcium signal in RGCs, we applied the T-type VGCC blocker mibefradil (3 µM), which reduced the signal by 21%±6% (p = 0.0011; n = 16; Fig. 7C). In addition, to show the extent to which Na channels contribute to the generation of [Ca^2+^]_i_ transients in RGC somata during the high K^+^ stimulation, we applied the Na channel blocker TTX. TTX (200 nM) reduced the calcium signal by 40%±9% (p = 0.0004; n = 14).

It is important to note that changes in the calcium signals of cells during VGCC block are not necessarily due only to block of the VGCCs in the RGCs themselves but may instead or in addition be due to block of VGCCs in a presynaptic cell that results in inhibition of the RGC calcium signal.

### Block of calcium signals induced by depolarization in RGC axons

**Figure 8:**
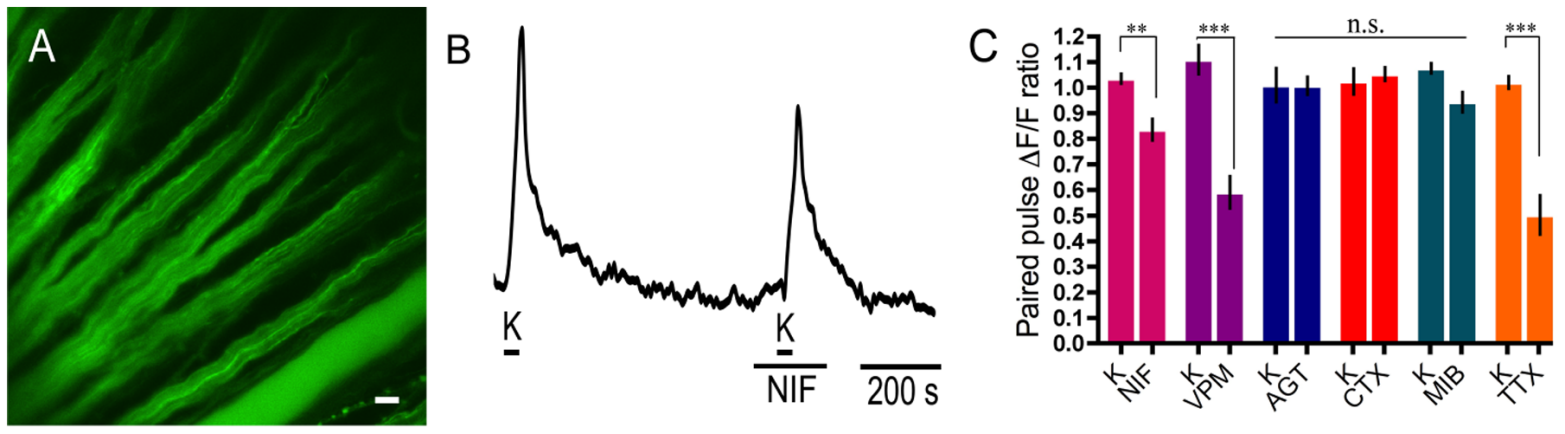
L-type VGCC subtypes contribute to calcium signalling in ganglion cell axons. A. Fluo-4 labelling of RGC axons in the wholemount retina. Scale bar is 20 µm. B. Application of nifedipine (NIF; 10 µM), an L-type Ca channel antagonist, reduced the second high K^+^-evoked calcium signal. C. Summary of Ca^2+^ imaging results from RGC axons showing the following changes in paired pulse Ca^2+^ signal in response to drugs (applied during the second K^+^ pulse) compared to their control paired K^+^ pulses (K): 10 µM nifedipine (20%±6%; p = 0.0053; n = 12), 100 µM verapamil (VPM; 52%±9%; p<0.0001; n = 9) and 200 nM TTX (52%±11%; p = 0.0009; n = 8). 400 nM ω-agatoxin IVA (AGT; n = 9), 3 µM ω-conotoxin-GVIA (CTX; n = 7) and 3 µM mibefradil (MIB; n = 12) did not change the calcium signal in a statistically significant manner.

Figure 8A shows fluo-4 labelling of the unmyelinated RGC axons on the surface of the retina. Calcium imaging of RGC axons demonstrated that superfusion of K^+^ (60 mM) elicited changes in mean fluorescence intensity with control paired K^+^ pulses typically producing a small pair-wise increase of ∼5%. To determine the contribution of L-type VGCCs to the depolarization-evoked [Ca^2+^]_i_, the L-type VGCC blockers nifedipine (10 µM) and verapamil (100 µM) were applied. Nifedipine and verapamil reduced the second of paired high K^+^ pulses in the RGC axons by 20%±6% compared to control (p = 0.0053; n = 12) and 52%±9% compared to control (p<0.0001; n = 9), respectively. The different degree of block of the calcium signal by verapamil and nifedipine could be due to the difference in concentration of the blockers and the different sensitivities of the Ca channels to these agents. To determine the contribution of P/Q- and N-type VGCCs to the calcium signal in axons, we applied ω-agatoxin IVA (400 nM) and ω-conotoxin-GVIA (3 µM), which did not reduce the Ca^2+^ signal in RGC and axons (Fig. 8C). The contribution of T-type VGCCs to the calcium signal in axons was tested with 3 µM mibefradil, which reduced the signal by 13%±8% but which was not a significant reduction (p = 0.13; n = 12). Figure 8C provides a summary of the effects of L-, P/Q-, N- and T-type VGCC blockers on calcium signals in RGC axons in the wholemount preparation.

Since TTX had reduced the calcium signal in RGC somata, we considered it important to confirm that action potentials generated in the RGC axons are a strong stimulus for VGCC activation. Applying the Na channel blocker TTX (200 nM) and measuring the change in peak calcium signal in response to high K^+^ depolarization, we found the calcium signal was reduced by 48%±11% (p<0.001; n = 8; Fig. 8), a similar reduction seen at the ganglion cell bodies.

We further compared the suppression of depolarization-evoked Ca^2+^ signals by TTX (200 nM) and mibefradil (3 µM) together in RGC somata and axons. When mibefradil and TTX were applied simultaneously to RGC somata, a block of 46%±10% compared to control was observed (p<0.0001; n = 22). When the reduction of Ca^2+^ signal in RGC axon bundles by mibefradil and TTX together was measured, a slightly larger mean reduction was observed compared to TTX alone, with a reduction of 55%±13% (p = 0.0004; n = 21) compared to control. In neither RGC somata nor their axon bundles was the mibefradil plus TTX reduction significantly different than the action of TTX alone.

## Discussion

The results presented here show strong correspondence between the immunohistochemical localization of Ca channel subunits in ganglion cells with the actions of Ca channel subtype blockers in the cell bodies and axons. The major findings are that RGC somata express L-, N-, P/Q-, and T-type Ca channels and that RGC axons express predominantly L-type Ca channels and that all of these channels subtypes provide robust calcium signalling contributions. The localization of T-type Ca channel α1 subunits in the RGC axons was not tested, but calcium imaging studies indicate that T-type Ca channels do not have a pronounced calcium signalling role in these structures. The results also highlight the strong stimulus provided by the firing of action potentials for the generation of calcium signals in both the cell bodies and the axons.

### VGCC localization to RGCs and RGC axons

These studies provide evidence for the differential localization of the α1 subunits that form L-, P/Q- and N-type VGCCs to the RGCs somata and their axons. Double immunostaining of RBPMS-labelled RGCs with antibodies against either the α1A, α1B, α1C, or α1D subunits, indicated the presence of these subunits in virtually all RGCs. Antibodies for α1C subunits also showed immunostaining in Müller cell processes, including their endfeet enveloping the RGCs in the GCL. Expression of the α1C subunit has been reported on amacrine and bipolar cells, as well as Müller cells in rat retina [33], [34]. Localization of the α1D subunit has been reported on Müller cells in rat [33] and AII amacrine cells in mouse [35] and ON-type bipolar cells in goldfish retina [36]. Further analysis to elucidate the localization of α1C and α1D to RGC dendrites is warranted.

Immunoreactivity for α1A, α1B, α1C, or α1D subunits was also localized to cell bodies in the GCL that did not show immunoreactivity for the RBPMS antibody. Due to the size of these somata (∼10 µm) and the specificity of RBPMS immunolabelling for RGCs [21], [22], these neurons are likely to be displaced amacrine cells [24], [25].

Co-immunolabelling with NF-M antibodies to identify RGC axon fibers showed the presence of α1B, α1C and α1D subunits in these axons. Triple labelling experiments with NF-M, RBPMS and α1A subunits antibodies failed in wholemounts. However, there was no evidence of axon fiber staining for α1A subunits in either wholemounts or sections.

Similar to previous findings that T-type Ca channels are not uniformly present in isolated RGCs [10], [11], [13], [28], we found that the occurrence of T channels in RGCs varied from about 30% in young rats to about 15% in adult rats. This frequency of finding T channels is partly explained by their preponderance in OFF RGCs recorded in intact retinal preparations [15], [16], and the age dependence is similar to previous reports showing increased T-channel activity in younger animals [13], [37]. Using whole cell patch clamp of isolated cells, we could not localize T-type Ca channels to cell structures other than the soma.

### Reduction of calcium signals by VGCC blockers

Analysis of calcium signalling in RGC somata performed in this work aligned with the immunohistochemical findings by providing evidence for L-, N-, P/Q- and T-type VGCCs in rat RGC somata. This conclusion arises from the reduction of depolarization-induced Ca^2+^ transients in the RGC soma by nifedipine and verapamil (L-type blockers), ω-conotoxin-GVIA (N-type blocker), ω-agatoxin-IVA (P/Q-type blocker), and the reducing trend of mibefradil (T-channel blocker). Previously, using patch clamp recording, the presence of L-type Ca channels in RGCs was shown with partial inhibition of the whole cell Ca channel current by nifedipine [11], [14]. The presence of N- and P/Q-type Ca channels was also previously noted with partial whole cell current inhibition by ω-conotoxin-GVIA and ω-agatoxin-IVA [11], [14]. A somatic role of R-type channels remains possible but is not easily demonstrated with calcium imaging. The presence of R-type Ca channels may be inferred by the remainder of whole cell Ca channel current resistant to blockers [11]. T-type Ca channels were previously identified in RGCs by their unique kinetic properties observed under voltage-clamp [10], [11], [15], and their preponderance in OFF ganglion cells, a subclass of RGCs [16], account for the incomplete occurrence of T-channels in our patch clamp identification.

VGCCs in cells presynaptic to RGCs could also drive calcium signals in RGCs that would be modified by VGCC blockers acting at these presynaptic sites. For example, L- and/or T-type Ca channels in bipolar cells mediate glutamate release [38]–[42], an excitatory influence on RGCs. In the presence of verapamil, nifedipine or mibefradil, reduced glutamate release from bipolar cells might lead to a reduction of RGC calcium signal. Block of N-, L-, or P/Q channels in amacrine cells should have the opposite effect since reduction of GABAergic or glycinergic inhibition onto RGCs or bipolar cell terminals could permit greater depolarization and increased activation of Ca channels in the RGCs [34], [35]. In addition, since TTX application would have reduced amacrine cell action potential generation, the indirect action of TTX inhibiting amacrine cells could also have led to greater depolarization of, and increased [Ca^2+^]_i_ signals in, RGCs. If any of the indirect effects were dominant, then the responses of ganglion cell bodies and axons would have shown similar patterns, which was the case for the L-type Ca channel blockers but not the N- and P/Q-type Ca channel blockers. In our experiments, these indirect calcium signal effects may well have been present but they were overridden by what appear to be the direct effects of Ca channel blockers and TTX on RGCs.

The calcium imaging analysis also provided evidence for L-type VGCCs in RGC axons, to the exclusion of other Ca channel subtypes. Block by nifedipine and verapamil was strong, but ω-conotoxin-GVIA, ω-agatoxin-IVA, and mibefradil did not reduce the Ca^2+^ signal significantly. These findings extend work indicating the presence of L-type Ca channels in myelinated rat optic nerve axons [43], albeit with limitations since that report used a combination of blockers that interact with numerous other ion channel types. In addition, an earlier report showed GABA-mediated modulation of N-type Ca channels in rat myelinated optic nerve [44], and indicated the lack of L-type Ca channels. There may be differences in the composition of activatable ion channels in myelinated and unmyelinated RGC axons, either due to protein trafficking or to post-translational modification, such as the degree of phosphorylation. Indeed, our immunohistochemical analysis indicated the presence of N-type Ca channels in unmyelinated axons while our calcium imaging experiments showed little or no N-type channel contribution to the axonal Ca^2+^ transients. Another complication arises from the potential for axon-glial signalling to have produced changes in axonal calcium levels during calcium channel block in astrocytes. Astrocytes abundantly express L-type Ca channels as well as most other VGCC types [45]. A reduction in their release of factors such as ATP induced by the L-type Ca channel blockers used here might influence axonal calcium levels.

### Role of calcium signalling in RGC axons

Compared to the well-established roles of Na and K channels in the generation of axonal action potentials, the roles of VGCCs are not as well-characterized or generalized in axons. Developmental roles [46] as well as dynamic tuning in the axonal initial segment [47] have been examined. Our results showing marked reduction of calcium signals in the presence of TTX, indicate that spike activity is a strong stimulus for raising [Ca^2+^]_i_ and leads to the suggestion that the amplitude of the calcium signal could record the frequency of action potential generation. Such a signal could be used by axonal trafficking systems to report the level of activity in an axon to the cell nucleus via trophic factors or other calcium-dependent signalling systems. This signalling might then direct protein synthesis and trafficking to the axon to support the changing needs for metabolic regulation. For example, our immunohistochemical localization of α1 Ca channel subunits in axons may reflect in part, trafficking of these proteins to the synaptic terminal where well-established roles for VGCCs exist in synaptic release.

### The role of Na channels in depolarization-induced Ca^2+^ signalling in RGCs

TTX blocks the generation and propagation of action potentials and was the strongest inhibitor of [Ca^2+^]_i_ during depolarization with 60 mM K^+^ in both RGC somata and their axons, an action that highlights the important stimulatory role of spikes in the activation of VGCCs. To determine whether the transient depolarizing nature of spikes had a preferential role in activating T-type Ca channels, we compared the actions of TTX, mibefradil, a T-type Ca channel blocker, and TTX and mibefradil together. Mibefradil by itself reduced Ca^2+^ transients relatively little, especially in axons, suggesting that the role of T-channels here is limited. Application of mibefradil plus TTX tended to reduce Ca^2+^ transients slightly more than TTX alone, but the increase was not significant. Mibefradil has been associated with block of other VGCCs and even Na channels in other tissues [48]–[50], but our results do not support the latter action strongly. Hence, block of action potentials is interpreted to reduce membrane depolarizations that activate all VGCCs in RGC somata and axons.

### Implications for injury and the role ofVGCCs blockers as potential therapy

Loss of vision caused by optic nerve damage and diseases such as glaucoma are characterized by RGC loss [51], [52]. Dysregulated calcium signalling causing RGC loss has been attributed to glutamate excitoxicity mediated by overstimulation of NMDA receptors [53], [54], compromised calcium buffering stores (for example, mitochondrial dysfunction) [55], excessive release of nitric oxide [56], and activation of Ca^2+^ permeable TRP channels [57]. While a mixture of channel blockers, including L- and T-type VGCC blockers, applied to the myelinated axons of the optic nerve abolished the immediate [Ca^2+^]_i_ increase following optic nerve crush and improved axon survivability [43], the detailed contribution of VGCCs to the calcium signal in the RGCs and their unmyelinated axons following injury remains unknown. Our results establish an understanding of VGCCs in RGCs to provide a platform for future experiments investigating their contribution in retinal injury models.

Many injuries, including traumatic brain injury (TBI), are thought to produce two phases of degradation, 1) a primary injury effect, which is characterized by rapid cellular degeneration, and 2) a secondary effect, characterized by a delayed response to the primary injury [58]. The secondary phase appears to be caused by calcium dysregulation [59] and has also been described as a ‘gradual and less massive increase in intracellular calcium [that may] become predictive of cell survival’ [4]. For these reasons the secondary phase is of particular interest due to the potential time window for the administration of therapeutic agents. VGCC blockers have been shown to ameliorate secondary RGC loss [60], [61].

Neurodegenerative diseases are characterized by a loss of axons as a result of trauma, excitotoxicity or ischemia [62], [63]. Therapeutic strategies to prevent cell death have focused on restoring homeostatic conditions within the neural soma, but have paid less attention to the axon. Emerging evidence suggests that neuronal cell damage can occur beyond the point of recovery earlier than it can be detected at the cell body and thus targeting the axon directly may produce more efficient and beneficial therapeutic strategies [64].

Our findings suggest that there could be a prominent role for VGCCs in the increase in calcium signal in damaged unmeylinated intraretinal axons, extending previous work that showed an increase in [Ca^2+^]_i_ following optic nerve crush *in vivo* [43]. This earlier work also demonstrated that application of a cocktail of L- and T-type Ca channel and AMPA receptor blockers to the myelinated axons of the optic nerve abolished the [Ca^2+^]_i_ increase following optic nerve crush. Since functional deficits can occur early in the progression of glaucoma and other optic neuropathies, targeted Ca channel blockade may have a role to play in how optic neurpathies are treated [65].

### Conclusion

The present findings establish the differential cellular distribution of VGCCs between RGC cell bodies and their unmyelinated axons. These results support a prominent role for L-type VGCCs in depolarization-induced increases in Ca^2+^ signals in RGC somata, and a role played solely by L-type VGCCs in unmeylinated intraretinal axons of RGCs. VGCC blockers provide a potential therapeutic strategy for the protection of RGCs and their axons following injury that can be rationalized by knowledge of the distribution of VGCCs expressed in RGCs compartments. Further understanding of the expression of specific VGCC subunits to the different subtypes of RGCs, as well as their dendrites and their myelinated and unmyelinated axons, is warranted.
